# PHLPP1 regulates CFTR activity and lumen expansion through AMPK

**DOI:** 10.1242/dev.200955

**Published:** 2022-08-23

**Authors:** Viola H. Lobert, Maren L. Skardal, Lene Malerød, Julia E. Simensen, Hermine A. Algra, Aram N. Andersen, Thomas Fleischer, Hilde A. Enserink, Knut Liestøl, Joan K. Heath, Tor Erik Rusten, Harald A. Stenmark

**Affiliations:** 1 Department of Molecular Cell Biology, Institute for Cancer Research, Oslo University Hospital, Montebello, Oslo 0379, Norway; 2 Centre for Cancer Cell Reprogramming, Faculty of Medicine, University of Oslo, Oslo 0379, Norway; 3 Department of Cancer Genetics, Institute for Cancer Research, Oslo University Hospital, Montebello, Oslo 0379, Norway; 4 Department of Informatics, University of Oslo, Oslo 0316, Norway; 5 Epigenetics and Development Division, Walter and Eliza Hall Institute of Medical Research, Parkville, Victoria 3052, Australia

**Keywords:** AMPK, CFTR, Epithelial, Lumenogenesis, PHLPP, 3D culture

## Abstract

Complex organ development depends on single lumen formation and its expansion during tubulogenesis. This can be achieved by correct mitotic spindle orientation during cell division, combined with luminal fluid filling that generates hydrostatic pressure. Using a human 3D cell culture model, we have identified two regulators of these processes. We find that pleckstrin homology leucine-rich repeat protein phosphatase (PHLPP) 2 regulates mitotic spindle orientation, and thereby midbody positioning and maintenance of a single lumen. Silencing the sole PHLPP family phosphatase in *Drosophila melanogaster*, *phlpp*, resulted in defective spindle orientation in *Drosophila* neuroblasts. Importantly, cystic fibrosis transmembrane conductance regulator (CFTR) is the main channel regulating fluid transport in this system, stimulated by phosphorylation by protein kinase A and inhibited by the AMP-activated protein kinase AMPK. During lumen expansion, CFTR remains open through the action of PHLPP1, which stops activated AMPK from inhibiting ion transport through CFTR. In the absence of PHLPP1, the restraint on AMPK activity is lost and this tips the balance in the favour of channel closing, resulting in the lack of lumen expansion and accumulation of mucus.

## Abstract (precis)

**Summary:** A human 3D culture model reveals that pleckstrin homology leucine-rich repeat phosphatase 1 is a previously unidentified regulator of the cystic fibrosis transmembrane conductance regulator channel.

## INTRODUCTION

Epithelial tubulogenesis typically forms a single lumen surrounded by a polarised epithelium. For several organs like the colon, this relies on fluid secretion secondary to apical ion transport, which results in lumen expansion. The chloride and bicarbonate channel cystic fibrosis transmembrane conductance regulator (CFTR) is the main driver of fluid transport in colon tubulogenesis (Navis and Bagnat, 2015). Mutations in CFTR are observed in the vast majority of individuals with cystic fibrosis (CF) and are responsible for clinical symptoms, which include respiratory and digestive problems due to the accumulation of thick and sticky mucus. CFTR localises to the apical domain of epithelial cells, where it secretes chloride and bicarbonate ions into the lumen in response to increasing intracellular cAMP, drawing sodium to create osmotic gradients and driving fluid movement. The volume and composition of this luminal fluid is essential for organ function. Mutations in CFTR that result in a defective CFTR lead to reduced luminal fluid as well as altered fluid composition, which are responsible for respiratory and digestive problems associated with CF (Riordan et al., 1989). Loss of CFTR function results in blocked lumen expansion in the salivary gland (Nedvetsky et al., 2014) and in Kupffer's vesicle (Navis et al., 2013), a fluid-filled structure lined with epithelial cells that express CFTR and is important for laterality in vertebrates (Essner, 2005). Increased chloride secretion due to enhanced CFTR activity is associated with pathologies such as cholera-induced enterotoxigenesis diarrhoea (Field et al., 1972), autosomal dominant polycystic kidney disease (ADPKD), which presents with enlarged cysts (Davidow et al., 1996), and hyperchloremia due to kidney dysfunction. It is therefore important to identify regulators of CFTR activity.

CFTR contains two membrane-spanning domains, two nucleotide-binding domains and a central regulatory (R) domain. Activity requires phosphorylation by cAMP-activated protein kinase A (PKA) at consensus sites in the R domain (Cheng et al., 1991), resulting in opening of the channel and ion secretion. Conversely, the secretion of chloride ions by CFTR is inhibited by phosphorylation by the energy sensor AMP-activated protein kinase (AMPK) at serine 768, resulting in closure of the channel and lack of chloride secretion (Hallows et al., 2003a,b, 2000; King et al., 2009; Kongsuphol et al., 2009). AMPK itself becomes phosphorylated at threonine 172 by LKB1 and calmodulin-dependent protein kinase kinases (CaMKKs) upon AMP binding (Towler and Hardie, 2007). However, how active AMPK is controlled in the context of chloride transport has not been established. Most recently, AMPK has been identified as a novel substrate for the phosphatase pleckstrin homology leucine-rich repeat protein phosphatase 1 (PHLPP1) (Behera et al., 2018), so we investigated whether PHLPP1 could regulate AMPK in the context of CFTR-driven lumen expansion.

In the Caco-2 3D model system, human colon cells are cultured in extracellular matrix and form a fluid-filled hollow sphere called cyst, similar to what is observed *in vivo*. This model presents the advantage of being able to study epithelial morphogenesis in the context of intestinal development *in vitro*. In this system, lumen position is defined during the first cell division. The apical membrane initiation site (AMIS) is established between the two dividing cells, at the site of the midbody. The midbody forms in the cleavage furrow as a last step during cytokinesis and has been shown to act as a polarity signal, determining whether the membrane develops an apical or basal character, and also directing the site of trafficking for further expansion of the central lumen (Lujan et al., 2017; Overeem et al., 2015). The cleavage furrow in the following cell divisions must therefore be asymmetrical with the midbody placed toward the apical membrane in the established central lumen (Lujan et al., 2017). Importantly, it is the position of the mitotic spindle that determines the position of the cleavage furrow. This structure forms between the two centrosomes and ensures faithful segregation of the chromosomes between the daughter cells. For the formation of a single lumen, the mitotic spindle must be oriented in parallel to the apical membrane, perpendicular to the apical-basal axis where it is anchored to the cortex during metaphase. Failure to orient the mitotic spindle properly can result in basolateral midbodies, giving rise to the formation of ectopic lumens (Bryant et al., 2010; Durgan et al., 2011; Hao et al., 2010; Hung et al., 2016; Jaffe et al., 2008; Qin et al., 2010). *In vivo*, the orientation of the mitotic spindle is important during asymmetric division of stem cells, which plays a role in determining cell fate (Chen et al., 2016).

The phosphatases pleckstrin homology leucine-rich repeat protein phosphatase PHLPP1 and PHLPP2 have mostly been characterised in the context of dephosphorylation of the serine/threonine kinase AKT, which is activated as a result of signalling through the phosphoinositide 3-kinase (PI3K) pathway (Brognard et al., 2007). AKT is involved in the regulation of metabolism, growth, survival, proliferation, angiogenesis and glucose uptake. As there are many more kinases in the genome than phosphatases, most phosphatases have more than one substrate. Consistent with this, other substrates of PHLPPs have been identified and include RAS (Shimizu et al., 2003) and PKC (Gao et al., 2008), as well as the mammalian sterile 20-like kinase 1 (MST1) (Qiao et al., 2010).

In this study, we show that PHLPP1 and PHLPP2 play different roles in the context of lumen development. Using a human intestinal 3D model, we find that PHLPP2 regulates mitotic spindle orientation and thereby midbody positioning and maintenance of a single lumen. Importantly, CFTR is the main channel regulating fluid transport in this system, and we find that CFTR activity is inhibited in the absence of PHLPP1. During lumen expansion, CFTR remains open through the action of PHLPP1, which stops activated AMPK from inhibiting ion transport through CFTR. In the absence of PHLPP1, the restraint on AMPK activity is lost and this tips the balance in the favour of channel closing, resulting in the lack of lumen expansion.

## RESULTS

### PHLPP1 and PHLPP2 depletion gives rise to different phenotypes

**Fig. 1.**
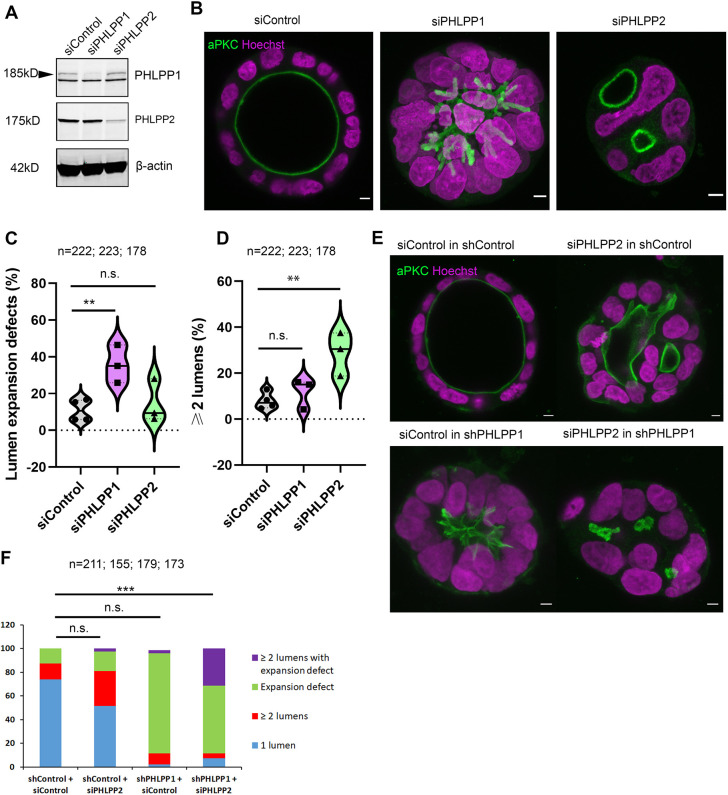
**PHLPP1 and PHLPP2 depletion gives rise to different phenotypes.** (A) Western blot analysis of Caco-2 lysates of cells transfected with siRNA targeting control (siControl), PHLPP1 (siPHLPP1) and PHLPP2 (siPHLPP2). (B) Caco-2 cells transfected with siControl, siPHLPP1 and siPHLPP2 were embedded in matrigel and collagen, and labelled using anti-aPKC (green) and Hoechst dye 33342 (magenta). Scale bars: 5 μm. (C) Violin plot showing percentage of cysts with lumen expansion defects for siControl-, siPHLPP1- and siPHLPP2-transfected Caco-2 cells. Median is indicated with a solid line; quartiles are indicated with dotted lines. **P<0.01 (two-way analysis of variance; siPHLPP1 compared with siControl). (D) Violin plot showing percentage of cysts with multiple lumens. Median is indicated with a solid line; quartiles are indicated with dotted lines. **P<0.01 (two-way analysis of variance; siPHLPP2 compared with siControl). (E) siPHLPP2 was transfected into Caco-2 cells expressing short hairpin RNA (shRNA) either non-targeting (shControl) or targeting PHLPP1 (shPHLPP1), embedded in matrigel and collagen, and labelled using anti-aPKC (green) and Hoechst dye 33342 (magenta). Scale bars: 5 μm. (F) Cysts were scored for one or at least two lumen expansion defects and multiple lumens with expansion defects. *n* represents the total number of Caco-2 cysts analysed and is indicated in each graph. ***P<0.001 (two-way analysis of variance; shPHLPP1+siPHLPP2 compared with shControl+siControl).

In an attempt to determine how PHLPP phosphatases regulate lumenogenesis, we first tested the effects of depleting PHLPP1 and PHLPP2 expression in Caco-2 colorectal adenocarcinoma cells (Fig. 1A,B). These cells develop into hollow spheres (cysts) surrounded by a single-layered polarised epithelium when cultured in extracellular matrix (Jaffe et al., 2008). Lumen formation was assessed by immunofluorescence and confocal microscopy using the apical marker aPKC. Lumens did not expand upon siRNA-mediated PHLPP1 depletion (Fig. 1B,C), and were rescued by stable low-level expression of siRNA-resistant GFP-PHLPP1 (Fig. S1A). The lack of lumen expansion was distinct from defects observed upon PHLPP2 depletion that instead caused the formation of multiple lumens (Fig. 1A,B,D and Fig. S1B). aPKC localisation remained unchanged, while E-cadherin was no longer exclusively at the plasma membrane upon PHLPP1 depletion (Fig. S1C). Importantly, depleting Caco-2 cells of both PHLPP1 and PHLPP2 gave rise to an additional category consisting of multiple lumens that were unable to expand (Fig. 1E,F). This was carried out in cells where PHLPP1 expression was depleted by shRNA, which produced an even more penetrant phenotype (Fig. 1F and Fig. S2A).

### PHLPP2 regulates mitotic spindle orientation and thereby single lumen formation

**Fig. 2.**
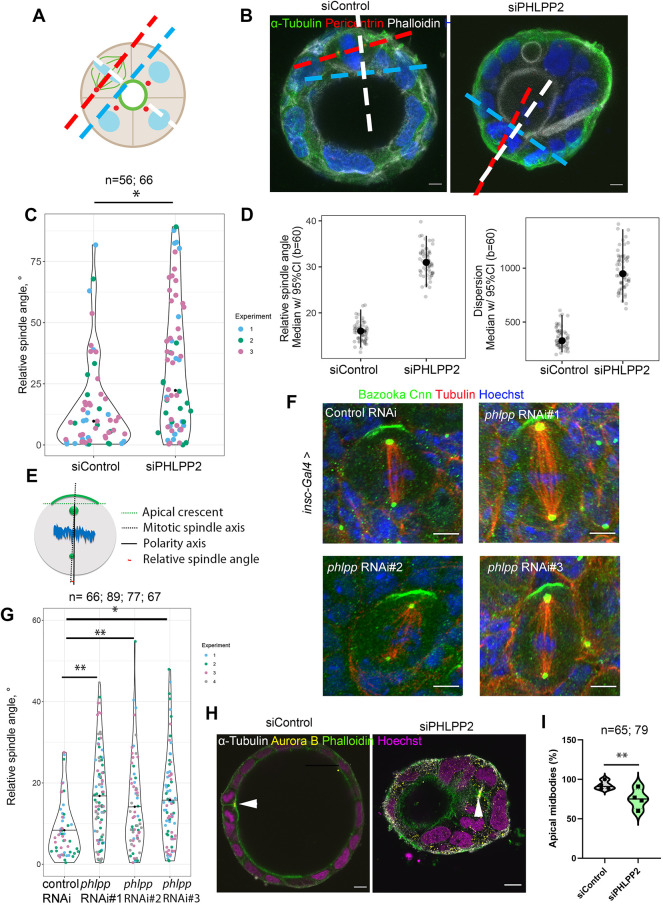
**PHLPP2 regulates mitotic spindle orientation in Caco-2 cysts and in *Drosophila melanogaster* neuroblasts, and single lumen formation.** (A) Schematic illustration of the calculation of mitotic spindle orientation in a metaphase cell of a Caco-2 cyst. It was measured by drawing a line at the apical membrane of the cyst (blue), a helper line perpendicular to the apical membrane (white) and a line through the centrosomes (red). Spindle angle was measured between the helper line and the mitotic spindle axis line. (B) Caco-2 cells transfected with siRNA targeting control or PHLPP2 were embedded in matrigel and collagen before labelling with anti-α-tubulin (green), anti-pericentrin (red) and phalloidin (white). The lines needed to calculate the spindle angle are drawn. Scale bars: 10 µm. (C) Violin plot presenting relative mitotic spindles angles in metaphase cells in Caco-2 transfected cysts. Median is indicated by a black dot. *P*=0.03 (two-way ANOVA comparing the two groups). *n* represents the total number of cysts. Three independent experiments were performed. (D) Bootstrapping was performed by resampling the empirical distributions of each parameters and computing the respective bootstrapped samples means a total of b=60 times. Means and 95% CI of relative angle and dispersion are indicated. (E) Schematic illustration of the calculation of the mitotic spindle orientation as the relative spindle angle (red line) between the polarity axis (black dashed line) drawn perpendicular to the apical crescent (green dashed line) and the mitotic spindle axis (black line) going through the two centrosomes. (F) Representative confocal images of control and *phlpp* RNAi #1, #2 and #3 neuroblasts stained using anti-Bazooka (green), anti-Cnn (green), anti-Tubulin (red) and Hoechst 33342 (blue). Scale bars: 5 µm. (G) Violin plot representing relative spindle angles in metaphase neuroblasts. Data were analysed using two-way analysis of variance **P<0.01 (control RNAi versus *Phlpp* RNAi #1), **P<0.01 (control RNAi versus *phlpp* RNAi #2) and *P<0.05 (control RNAi versus *phlpp* RNAi #3). The mean is indicated by a solid line. Individual spindle angles are presented as dots and four independent experiments were performed. (H) Caco-2 cells transfected with siRNA targeting control or PHLPP2 were embedded in matrigel before labelling using anti-aurora B (yellow), anti-α-tubulin (white) and phalloidin (magenta). White arrowheads indicate the midbodies. Scale bars: 10 µm. (G) Violin plot showing the percentage of apical midbodies in Caco-2 cysts transfected with siControl or siPHLPP2. The median is indicated with a solid line. **P<0.01 (χ^2^ test in a three-way contingency table analysis comparing siControl and siPHLPP2). Four independent experiments were performed.

As mitotic spindle orientation defects are known to result in multiple lumens, we investigated whether the absence of PHLPP2 affects spindle orientation (Fig. 2A-D). We observed that, in control cysts, aberration from the theoretical angle of 90° was close to zero, whereas without PHLPP2, spindle angle was much more heterogenous (Fig. 2C). Next, we investigated the role of Phlpp *in vivo* in *Drosophila melanogaster* larval neuroblasts, an established model to study mitotic spindle orientation (Morin and Bellaïche, 2011). Notably, there is only one Phlpp in *Drosophila*. In this system, the mitotic spindle aligns itself along the apico-basal polarity axis (Fig. 2E). As indicated, the relative spindle angle was calculated as the difference between the spindle axis (spanning through the centrosomes, dashed line) and the polarity axis (perpendicular to the apical Bazooka crescent) (Fig. 2E). *phlpp* RNAi was driven specifically in neuroblasts using the driver inscuteable (Fig. 2F). The distribution of relative mitotic spindle angles in Phlpp-deficient metaphase neuroblasts was significantly different from the angles measured in control neuroblasts (Fig. 2F,G).

Next, we investigated whether mislocalised midbodies were present in the absence of PHLPP2, as this has been described as a consequence of misoriented mitotic spindles (Lujan et al., 2017; Overeem et al., 2015). The site of abscission and midbody positioning during cytokinesis defines the apical membrane initiation site and, in the subsequent cell division, abscission must be asymmetrical to maintain a single lumen. The midbody must therefore be placed towards the apical membrane when the central lumen has been established (Li et al., 2014a,b; Wang et al., 2014) (Fig. 2H). Consistent with this, 92% of midbodies in control cysts were observed immediately below the apical membrane (detected by Aurora B staining), whereas only 72% of midbodies localised apically upon PHLPP2 depletion (Fig. 2H,I). Therefore, PHLPP2 is required for correct mitotic spindle orientation, which may lead to apical midbodies and single lumen formation.

### PHLPP1 regulates lumen expansion through CFTR

**Fig. 3.**
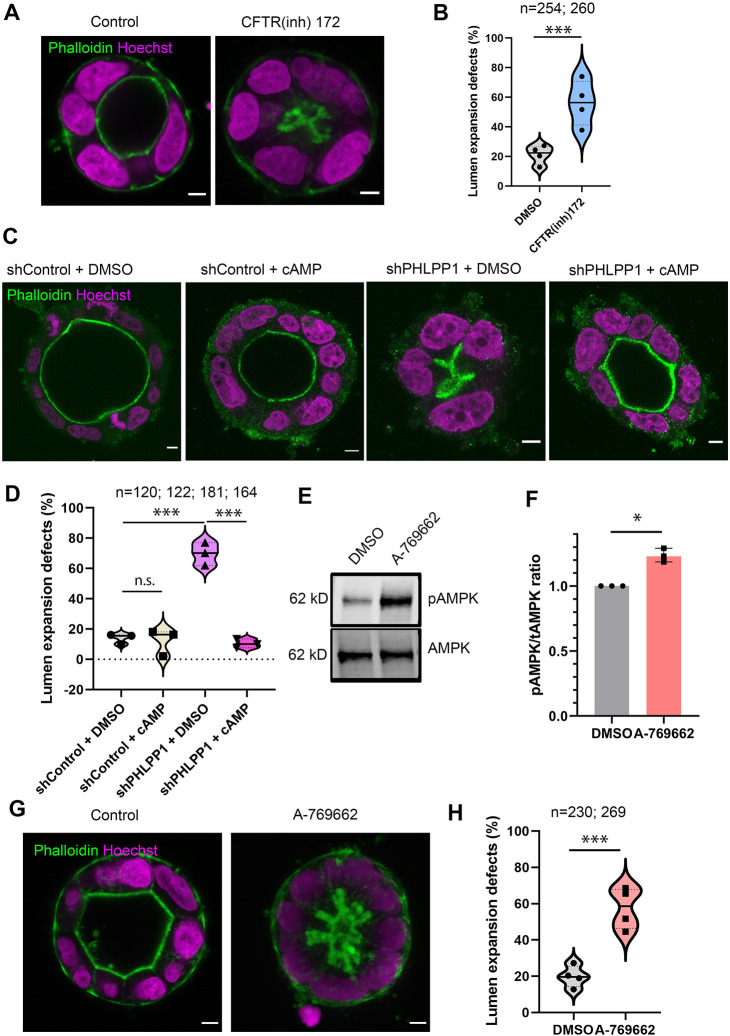
**PHLPP1 regulates lumen expansion through CFTR.** (A) Caco-2 cells incubated with 50 μM CFTR inhibitor (CFTRinh-172) were embedded in matrigel and collagen for 72 h and labelled using phalloidin (green) and Hoechst dye 33342 (magenta). Scale bars: 5 μm. (B) Violin plot showing the percentage of CFTRinh-172-treated cysts with lumen expansion defects. Median is indicated with a solid line; quartiles are indicated with dotted lines. ***P<0.001 (one-way analysis of variance comparing DMSO with CFTR inhibitor). Four independent experiments were performed. (C) Caco-2 cells expressing short hairpin RNA (shRNA) either non-targeting (shControl) or targeting PHLPP1 (shPHLPP1) were incubated with 100 µM of the cAMP analogue 6-Bnz-cAMP for 72 h before labelling using phalloidin (green) and Hoechst 33342 (magenta). Scale bars: 5 μm. (D) Violin plot showing the percentage of shControl and shPHLPP1 cAMP-treated cysts with lumen expansion defects. Median is indicated with a solid line; quartiles are indicated with dotted lines. ***P<0.001 (one-way analysis of variance comparing shPHLPP1 + DMSO with shControl + DMSO and comparing shPHLPP1+cAMP with shPHLPP1 + cAMP). Data are mean three independent experiments. (E) Western blot analysis of Caco-2 cells incubated with 30 μM of the AMPK activator A-769662 and immunoblotted with antibodies against phospho-AMPK and AMPK. (F) Graph showing the mean phospho-AMPK/AMPK ratio determined from relative signal intensities of phospho-AMPK and AMPK bands after 2 h treatment with 30 μM of A-769662. *P<0.05 (one sample paired *t*-test on log values comparing treatment with DMSO). Control values were normalised to 1 and the data points representing the mean of three independent experiments were plotted. Error bars represent 95% CI. (G) Caco-2 cells incubated with 30 μM of A-769662 were embedded in matrigel and collagen for 72 h, and labelled using phalloidin (green) and Hoechst dye 33342 (magenta). Scale bars: 5 μm. (H) Violin plot presenting the percentage of AMPK activator-treated cysts with lumen expansion defects. Median is indicated with a solid line; quartiles are indicated with dotted lines. ***P<0.001 (one-way analysis of variance comparing DMSO with A-769662). *n* represents the total number of Caco-2 cysts evaluated and is indicated in the graphs.

As CFTR is the main ion channel in the colon, where it regulates fluid filling (Dekkers et al., 2013), we first tested whether lumen expansion in Caco-2 colorectal adenocarcinoma cells was dependent on CFTR. Incubation with a CFTR inhibitor (CFinh-172) led to lumen expansion defects (Fig. 3A,B and Fig. S2B), validating the Caco-2 cell line as a model for CFTR-driven luminal filling. cAMP stimulates PKA-dependent fluid filling by activation of CFTR (Jaffe et al., 2008). Incubation of shPHLPP1 cells with the cAMP analogue N6-benzoyl-cAMP (6-Bnz-cAMP), which selectively activates PKA, restored lumen expansion (Fig. 3C,D and Fig. S3A), suggesting that PHLPP1 regulates CFTR activity. Importantly, we could show that CFTR localisation remained apical upon PHLPP1 depletion, confirming that a trafficking defect is not responsible for the lumen expansion defect observed (Fig. S3B).

### PHLPP1 facilitates CFTR activity through AMPK

AMPK has been found to be dephosphorylated at Thr172 by PHLPP1 (Behera et al., 2018). We therefore asked whether PHLPPs might regulate AMPK in the context of lumen expansion, as AMPK is a known negative regulator of CFTR activity (Hallows et al., 2003a,b, 2000). We first investigated whether activating AMPK acted as a negative regulator of CFTR in Caco-2 cysts. The AMPK activator A-769662 activates AMPK by allosteric activation and inhibition of dephosphorylation (Göransson et al., 2007), and incubation of Caco-2 cells with A-769662 caused elevated levels of phospho-AMPK (Fig. 3E,F) and produced an increase in lumen expansion defects (Fig. 3G,H and Fig. S3C), suggesting that increased AMPK activity inhibits lumen expansion.

**Fig. 4.**
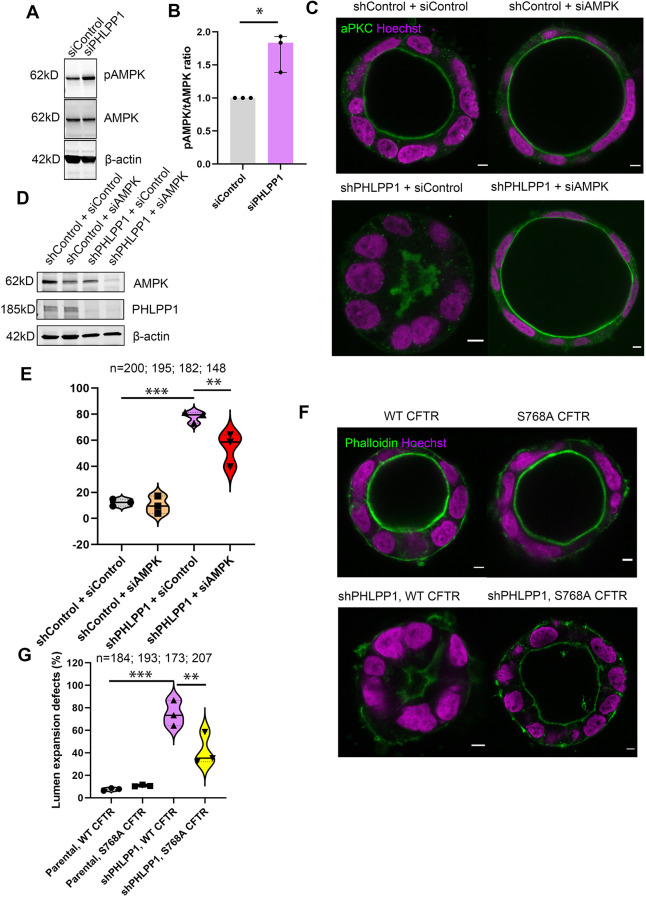
**PHLPP1 regulates CFTR activity through AMPK.** (A) Western blotting of Caco-2 cells depleted of PHLPP1 and immunoblotted using antibodies against phospho-AMPK, AMPK and β-actin. (B) Graph showing the median phospho-AMPK/AMPK ratio determined from relative signal intensities of phospho-AMPK and AMPK bands following depletion of PHLPP1 by siRNA. *P<0.05 (one sample *t*-test comparing siPHLPP1 with siControl). Control values are normalised to 1. Error bars represent 95% CI. (C) Caco-2 cells expressing short hairpin RNA (shRNA) either non-targeting (shControl) or targeting PHLPP1 (shPHLPP1) were transfected with either siControl or siRNA targeting AMPK before being embedded in matrigel and collagen. Cysts were labelled using anti-aPKC (green) and Hoechst 33342 dye (magenta). Scale bars: 5 μm. (D) Western blotting of shControl and shPHLPP1 cells depleted of AMPK and immunoblotted with antibodies against AMPK, PHLPP1 and β-actin. (E) Violin plot presenting the percentage of cysts with lumen expansion defects. Median is indicated with a solid line; quartiles are indicated with dotted lines. ***P<0.001 (one-way analysis of variance comparing shPHLPP1+siControl with shControl+siControl); **P<0.005 (one-way analysis of variance comparing shPHLPP1+siAMPK with sPHLPP1+siControl. (F) Control or shPHLPP1 Caco-2 cells stably expressing CFTR WT or CFTR S768A were embedded in matrigel and collagen, and labelled using phalloidin (green) and Hoechst 33342 dye (magenta). Scale bars: 5 μm. (G) Violin plot showing penetrance of the lumen expansion defects. Median is indicated with a solid line; quartiles are indicated with dotted lines. ***P<0.001 (one-way analysis of variance comparing parental WT CFTR with shPHLPP1 WT CFTR) and **P<0.01 (one-way analysis of variance comparing shPHLPP1 WT CFTR with shPHLPP1 S768A CFTR). Three independent experiments were performed. *n* represents the total number of Caco-2 cysts evaluated and is indicated in the graphs.

As PHLPP1 depletion phenocopied CFTR inhibition and AMPK activation, we hypothesised that PHLPP1 might regulate CFTR through AMPK (Behera et al., 2018). In Caco-2 cells, we observed increased phospho-AMPK at Thr172 upon depletion of PHLPP1 (Fig. 4A,B), suggesting that AMPK activity is indeed increased in the absence of PHLPP1, and could be directly dephosphorylated by PHLPP1. In order to investigate whether the regulation of lumen expansion by AMPK might be controlled by PHLPP1, we co-depleted AMPK and PHLPP1 in Caco-2 cells. Importantly, co-depletion of AMPK resulted in a partial rescue of the inhibitory effect on lumen expansion caused by PHLPP1 depletion (Fig. 4C-E and Fig. S3D-F). This suggests that AMPK hyperactivation when PHLPP1 is depleted is the cause of CFTR filling defects. To test this, we made stable cell lines expressing wild-type CFTR or CFTR S768A, which cannot be phosphorylated by AMPK (Hallows et al., 2000). Expression of CFTR S768A in PHLPP1-depleted cells partially rescued the lumen expansion defect (Fig. 4F,G and Fig. S4B). We therefore conclude that PHLPP1 facilitates lumen expansion through AMPK, thereby inhibiting the ability of AMPK to phosphorylate CFTR and restrict luminal filling.

### Loss of PHLPP1 results in MUC2 accumulation

**Fig. 5.**
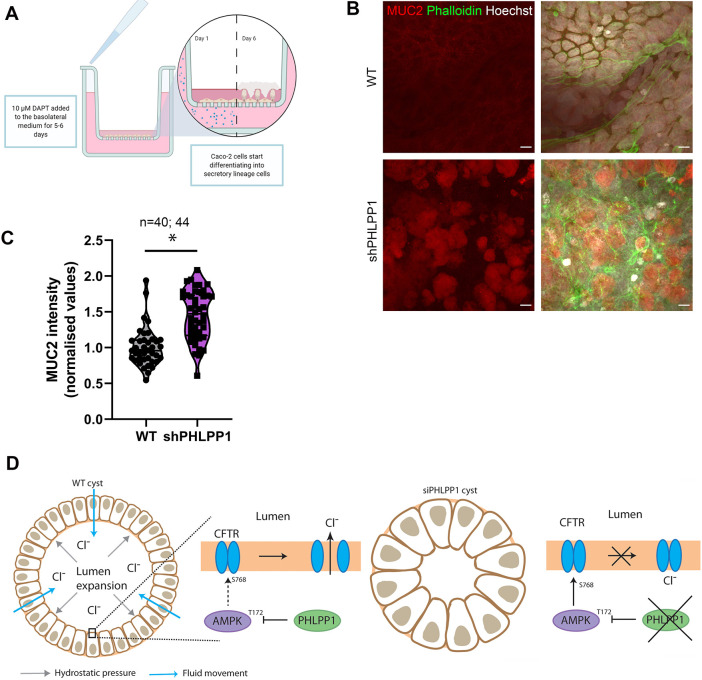
**Loss of PHLPP1 results in accumulation of MUC2.** (A) Schematic illustration of the protocol use to differentiate enterocytes into mucus-producing goblet cells. (B) Caco-2 cells treated with DAPT combined with semi-wet interface and mechanical stimulation stably expressing shControl or shPHLPP1 were labelled with anti-MUC2 (red), phalloidin (green) and Hoechst 33342 dye (white). Scale bars: 10 µm. (C) Violin plot presenting MUC2 intensity values. Four independent experiments were performed. *P<0.05 (two-way analysis of variance comparing shPHLPP1with to shControl). Individual intensity measurements are plotted. (D) Cartoon model. Lumen expansion relies on CFTR-mediated transport of chloride and bicarbonate over the cell membrane to allow fluid filling. Under normal conditions, PHLPP1 restrains active pAMPK (pT172) levels, allowing CFTR-mediated transport of chloride and bicarbonate to the lumen. In the absence of PHLPP1, active AMPK accumulates. This leads to more inhibitory phosphorylation of CFTR and reduced chloride and bicarbonate transport, thereby causing lumen expansion defects and mucus accumulation (not depicted). For simplicity, bicarbonate is not indicated in the figure.

As inactive CFTR results in the accumulation of sticky mucus in individuals with CF (Boucher, 2007), we investigated whether loss of PHLPP1 would result in the accumulation of mucins in a system containing mucus-producing cells. Caco-2 cells, which are enterocytes derived from a colorectal adenocarcinoma, were differentiated into mucus-producing goblet cells using the γ-secretase inhibitor *N*-[(3,5-difluorophenyl)acetyl]-l-alanyl-2-phenyl]glycine-1,1-dimethylethyl ester DAPT as well as semi-wet interface combined with mechanical stimulation (Quintana-Hayashi and Lindén, 2018) (Fig. 5A). This gave rise to an epithelium with a layer of mucus that was rich in mucin 2 (MUC2). We observed that loss of PHLPP1 resulted in accumulation of MUC2 in extracellular patches above the polarised epithelium, consistent with the proposed role in regulating CFTR activity (Fig. 5B,C). Thus, mucus accumulates in the absence of PHLPP1, consistent with the fact that PHLPP1 regulates CFTR activity (Fig. 5D).

## DISCUSSION

Although lumen formation and expansion play crucial roles during development, surprisingly few regulators of these processes have been identified. In this study, we identify the phosphatase PHLPP1 as a required factor for lumen expansion in Caco-2 cysts, whereas PHLPP2 regulates single lumen formation through mitotic spindle orientation in Caco-2 cysts and *Drosophila melanogaster* neuroblasts. We find that PHLPP1 regulates lumen expansion through regulation of CFTR activity by dephosphorylating AMPK. Although the dephosphorylation of AMPK by PHLPP1 has been established by others (Behera et al., 2018), we identify here a biological role for this regulation in the context of CFTR activity and lumen expansion. As PHLPP1 facilitates chloride secretion, inhibitors of PHLPP1 may prove to be useful in the treatment of ADPKD, where activators of AMPK have already been shown to slow renal cystogenesis (Takiar et al., 2011). Interestingly, AMPK has emerged as an important regulator of several other ion channels, not only CFTR (Andersen and Rasmussen, 2012), suggesting that PHLPP1 could act as a regulator of AMPK in the context of other ion channels that are expressed in other cell types/organs. Importantly, CFTR also transports bicarbonate, which is known to play a role in the release of mucus (Chen et al., 2010) and could explain the accumulation of mucus observed in the absence of PHLPP1. Our results therefore suggest that PHLPP1 plays an important role in lumenogenesis through its regulation of CFTR and thin mucus.

## MATERIALS AND METHODS

### Reagents and antibodies

PHLPP1 antibody from Proteintech (1:500, 22789-1-AP-20) was used for western blotting (Baffi et al., 2019). PHLPP2 antibody was purchased from Bethyl (1:500; A300-661A). Other antibodies used included anti-aPKC (1:500; sc-216, Santa Cruz Biotechnology), anti-β-actin (1:10,000; A5316, Sigma-Adrich), anti-p-AMPK (1:200; 2535, Cell Signaling Technology), anti-AMPK (1:200; 2793S, Cell Signaling Technology) and anti-MUC2 (1:100; sc-515032, Santa Cruz Biotechnology), anti-Aurora B (1:500, AB2254; Abcam). Rabbit anti-Cnn (1:500) was kindly provided by Thomas C. Kaufman (Indiana University, Bloomington, USA). Sheep anti-α/β-tubulin (1:200; ATN02) was purchased from Cytoskeleton. Rabbit anti-Bazooka (1:500) was obtained from Tony Harris (University of Toronto, Canada) (Siller et al., 2006). Fluorescently labelled phalloidin (phalloidin 488 used at 1:500) was obtained from Molecular Probes, while secondary antibodies included Alexa Fluor 488 donkey anti-mouse (1:500; 715-545-151, Jackson ImmunoResearch), Alexa Fluor 488 donkey anti-rabbit (1:500; 711-545-152, Jackson ImmunoResearch), Alexa Fluor 568 donkey anti-mouse (1:500; A10037, Molecular Probes), Alexa Fluor 568 donkey anti-rabbit (1:500; A10042, Molecular Probes), Alexa Fluor 647 donkey anti-mouse (1:200; 715-605-150, Jackson ImmunoResearch). For western blotting, secondary antibodies used were donkey anti-rabbit IRDye 800CW (1:5000; Li-Cor, 926-32213), IRDye 800CW donkey anti-mouse IgG (H+L) (1:5000; Li-Cor, 926-32212), donkey anti-mouse IRDye 680 (1:5000; Li-Cor, 926-32222) and donkey anti-rabbit IRDye 680RD (1:5000; Li-Cor, 926-68073). 6-Bnz-cAMP was purchased from Sigma; Matrigel was from Corning. The CFTR inhibitor CFinh-172 was obtained from the Cystic Fibrosis Foundation. The AMPK activator A-769662 was purchased from Cayman. Wild-type CFTR and CFTR S768A were kind gifts from Kenneth Hallows (Keck School of Medicine, University of Southern California, USA).

### Cell culture, siRNA transfection and 3D culturing

Caco-2 cells were purchased from American Type Culture Collection (ATCC) and authenticated on 12.03.2019 at 100% match by ATCC. Cell lines were routinely tested for mycoplasma contamination. Caco-2 cells were routinely cultured in DMEM at 37°C and 5% CO_2_ supplemented with 15% FCS, 100 U ml^−1^ penicillin and 1 μg ml^−1^ streptomycin (Gibco). For siRNA studies, 8×10^5^ cells were seeded into 6 cm dishes 1 day before transfection. Transfection was performed using Lipofectamine RNAi Max (Invitrogen) according to the manufacturer's specifications. One day post-transfection, cells were embedded in matrigel and collagen for a period of 5 days, as previously described (Jaffe et al., 2008), unless otherwise specified. The siRNAs used have the following sequences: scrambled, 5′-UAGCGACUAAACACAUCAA-3′; PHLPP1, 5′-UGUAGAAUAUGGAGACUAA-3′; PHLPP2, 5′-GAACUUGUCCCAUAAUU-3′. AMPK siRNA was purchased from Santa Cruz (sc-29673).

### Immunofluorescence and confocal microscopy

Cysts and transwells were fixed with 4% methanol-free formaldehyde for 60 min at room temperature. Cysts were washed and permeabilised by three 20 min washes in wash buffer (1.3 M NaCl, 132 mM Na_2_HPO_4_, 35 mM NaH_2_PO_4_, 75 mM NaN_3_, 0.5% BSA, 2% Triton X-100, 0.4% Tween-20). Transwells were permeabilised in 0.3% Triton X-100 for 30 min and blocked in 5% BSA containing 15% FCS for 1 h. Next, cysts and transwells were incubated with primary antibodies diluted in 3% BSA in PBS overnight, followed by a 2 h secondary antibody incubation, and counterstained with the DNA dye Hoechst 33342 and Alexa Fluor 488/647 phalloidin. Confocal images were acquired on a Zeiss LSM 710, 780 or 880 confocal laser-scanning microscope. At least ten images were captured for each representative image depicted in the figures, and a minimum of three experiments was performed (*n* indicated in each figure).

### Generation of stable cell lines

GFP-tagged PHLPP1, GFP-tagged CFTR and GFP-tagged S768A were generated as Gateway pENTR-GFP by conventional restriction enzyme-based cloning using the primers GAATTCGGGCGGCCGCaaATGCAGAGGTCGCCTCTGG and GGTCTAGAGCTCGAGCTAAAGCCTTGTATCTTGCACCTCTTC. A lentiviral transfer vector was generated by recombination into pLenti destination vector, using Gateway LR reaction (Invitrogen). Lentiviral particles were packaged using a third generation packaging system (Dull et al., 1998). Caco-2 cells were transduced with lentiviral transduction particles containing shRNA targeted to PHLPP1 transcripts (Sigma, 82793), GFP-PHLPP1, GFP-CFTR or S768A GFP-CFTR; stable cell populations were selected by antibiotic selection (GFP-PHLPP1) or by flow cytometry (GFP-CFTR, S768A CFTR).

### Measurement of mitotic spindle orientation in Caco-2 cysts

Mitotic spindle angles were measured by making a line at the apical membrane (phalloidin-positive), a helper line (perpendicular to the apical membrane) and a line through the centrosomes (spindle axis) in metaphase cells. The angle between the helper line and mitotic spindle axis was measured using Image J. Relative spindle angles, defined as the difference between the theoretical angle (90°) and the measured mitotic spindle angle, was calculated.

### *Drosophila* stocks and RNAi-mediated silencing of Phlpp in neuroblasts

All fly stocks were maintained and fly crosses were performed at 25°C. Control RNAi flies (*y^1^v^1^ ;attP2*, 36303) and *phlpp* RNAi#1 flies (P{TRiP.HMC04703}attP40) were from Bloomington Drosophila Stock Centre (BDSC, Indiana University, USA), whereas *phlpp* RNAi#2 (P{GD6992}v45363, 45363) and *phlpp* RNAi#3 (P{KK100988}VIE-260B, 110360) were obtained from Vienna Drosophila Resource Centre [VDRC, Vienna Biocenter Core Facilities (VBCF), Austria]. Knockdown in NBs was performed by crossing virgins of the NB driver line UASDicer-2;insc-Gal4, UAS-CD8::GFP [kindly provided by Jürgen A. Knoblich, Institute of Molecular Biotechnology of the Austrian Academy of Sciences (IMBA), Vienna, Austria] (Neumüller et al., 2011) to control or *phlpp*-RNAi males.

### Mitotic spindle orientation in neuroblasts

This was measured as previously described (Malerød et al., 2018). Brains of third-instar larvae in the food were dissected 4 days after egg lay, fixed (4% formaldehyde in PBS-0.1% Triton X-100 for 20 min at room temperature) and stained with primary antibodies against Cnn, Bazooka and α/β-Tubulin (4°C, overnight). After repeatedly washing (three times for 20 min in PBS-0.1% Triton X-100), the brains were incubated with secondary antibodies and 1 µg/ml Hoechst 33342 (2 h, room temperature), washed (three times for 20 min in PBS-0.1% Triton X-100), mounted in Vectashield (Vector Laboratories) and examined by confocal fluorescence microscopy (Zeiss LSM 780). The mitotic spindle orientation, defined as the relative spindle angle between the spindle axis (going through the two centrosomes) and polarity axis (perpendicular to the line aligning the apical Bazooka crescent) was calculated using ImageJ.

### Semi-wet interface with mechanical stimulation

Enterocytes were differentiated into mucus-producing cells using *N*-[(3,5-Difluorophenyl)acetyl]-L-alanyl-2-phenyl]glycine-1,1-dimethylethyl ester (DAPT) treatment combined with a semi-wet interface and mechanical stimulation as described by others (Quintana-Hayashi and Lindén, 2018). Briefly, cells were seeded out onto 12 mm transwells with 0.4 µm pores (Corning) and left until they reached confluency. 10 µM DAPT was added to the basolateral compartment for a period of 5 days (changed daily) in order to induce differentiation of the cells. Medium was then removed from the apical compartment to create a semi-wet interface, and this was combined with constant rocking for a period of 22 days. Basolateral medium was replaced three times a week, and the apical medium was kept at a constant volume of 100 µl.

### Statistical analysis

Two-way analysis of variance was used to obtain *P*-values when comparing lumen phenotypes resulting from knockdown treatment to controls. One-way analysis of variance was used to obtain *P*-values when comparing lumen phenotypes resulting from pharmacological treatment to control. The statistical tests performed for each experiment are specified in the figure legends. *P*-values are indicated for each experiment.

## Supplementary Material

Supplementary information

Reviewer comments
