# Supplementary material for: PHLPP1 regulates CFTR activity and lumen expansion through AMPK
Source: Development. 2022 Aug 23;149(20):dev200955. doi: 10.1242/dev.200955 (PMC9534488; doi:10.1242/dev.200955)
Supplement: Supplementary information [file develop-149-200955-s1.pdf]

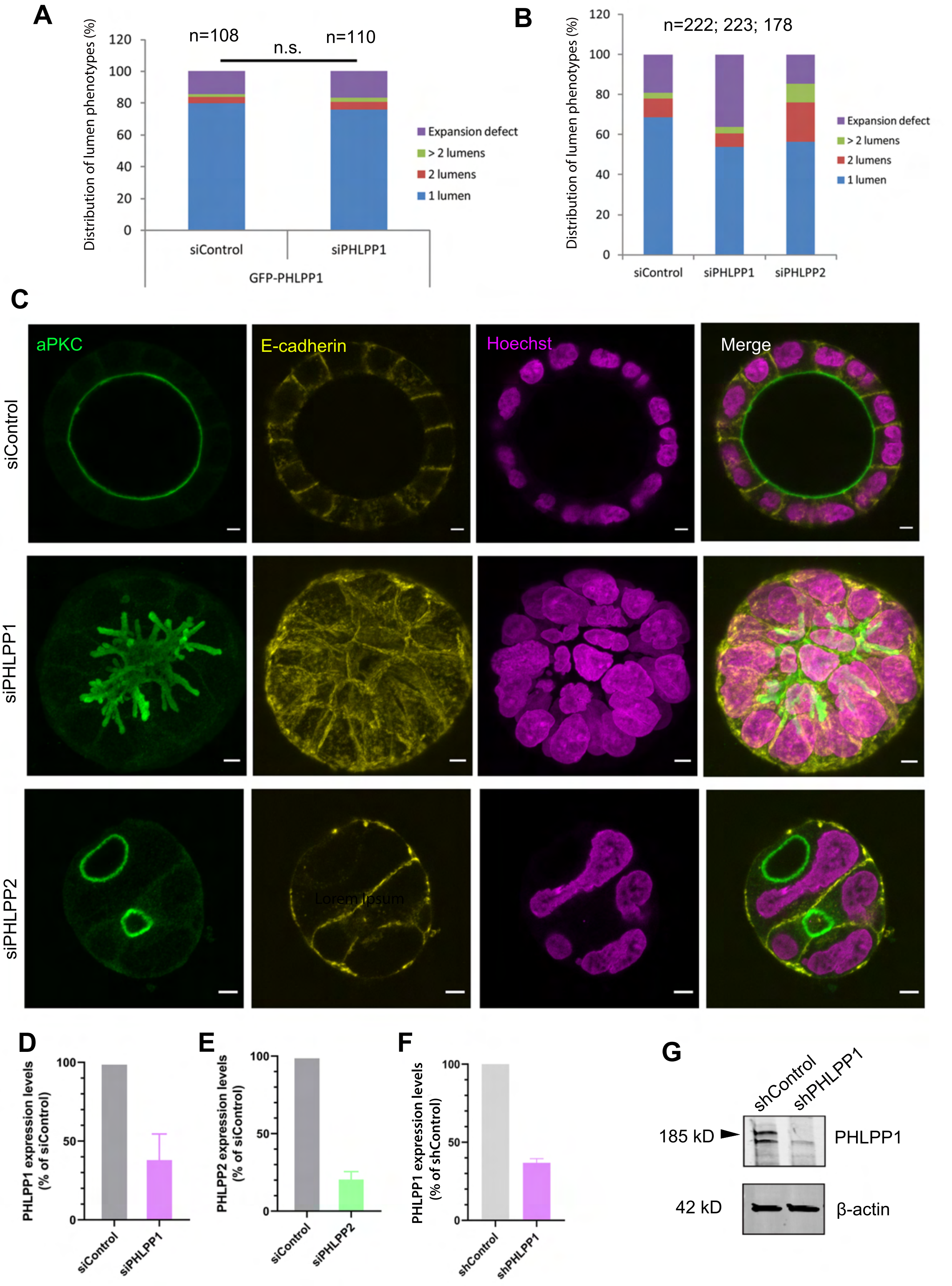

A
Distribution of lumen phenotypes (%)
120
100
80
60
40
20
0
n=108
n.s.
n=110
Expansion defect
> 2 lumens
2 lumens
1 lumen
siControl
siPHLPP1
GFP-PHLPP1
B
Distribution of lumen phenotypes (%)
n=222; 223; 178
siControl
siPHLPP1
siPHLPP2
C
aPKC
E-cadherin
Hoechst
Merge
siControl
siPHLPP1
siPHLPP2
Lorem Ipsum
D
PHLPP1 expression levels (% of siControl)
100
50
0
siControl
siPHLPP1
E
PHLPP2 expression levels (% of siControl)
siPHLPP2
F
PHLPP1 expression levels (% of shControl)
shControl
shPHLPP1
G
shControl
shPHLPP1
185 kD
PHLPP1
42 kD
β-actin

Fig. S1. (A) GFP-PHLPP1 cells transfected with siRNA targeting PHLPP1 were embedded in matrigel and scored for lumen categories 1, 2, ≥2 and lumen expansion defects. The siRNA targets the 3'UTR so overexpression of GFP-PHLPP1 is siRNA-resistant. One-way analysis of variance *p*-value comparing siPHLPP1 to siControl in PHLPP1-GFP cells was 0.62. *n* represents the total number of Caco-2 cysts evaluated in three independent experiments and is indicated in the graphs. (B) Caco-2 cells transfected with siRNA targeting Control, PHLPP1 and PHLPP2 were grown as cysts and scored as in (A). *n* represents the total number of Caco- 2 cysts evaluated in three independent experiments and is indicated in the graphs. (C) Caco-2 cells transfected with siRNA targeting Control, PHLPP1 and PHLPP2 were labelled with anti-aPKC (green), anti-E-cadherin (yellow) and Hoechst dye 33342 (magenta). Scale bar, 5µm. (D- F) Graph showing the median of protein expression levels determined from relative signal intensities of PHLPP1 or PHLPP2 and β-actin bands following depletion of PHLPP1 or PHLPP2 by siRNA (D,E), or by stable expression of shPHLPP1 (F). Control values were normalised to 1. Error bars represent s.e.m. (G) Western blotting of Caco-2 cells expressing short hairpin RNA (shRNA) either non-targeting (shControl) or targeting PHLPP1 (shPHLPP1) and immunoblotted with antibodies against PHLPP1 and β-actin.

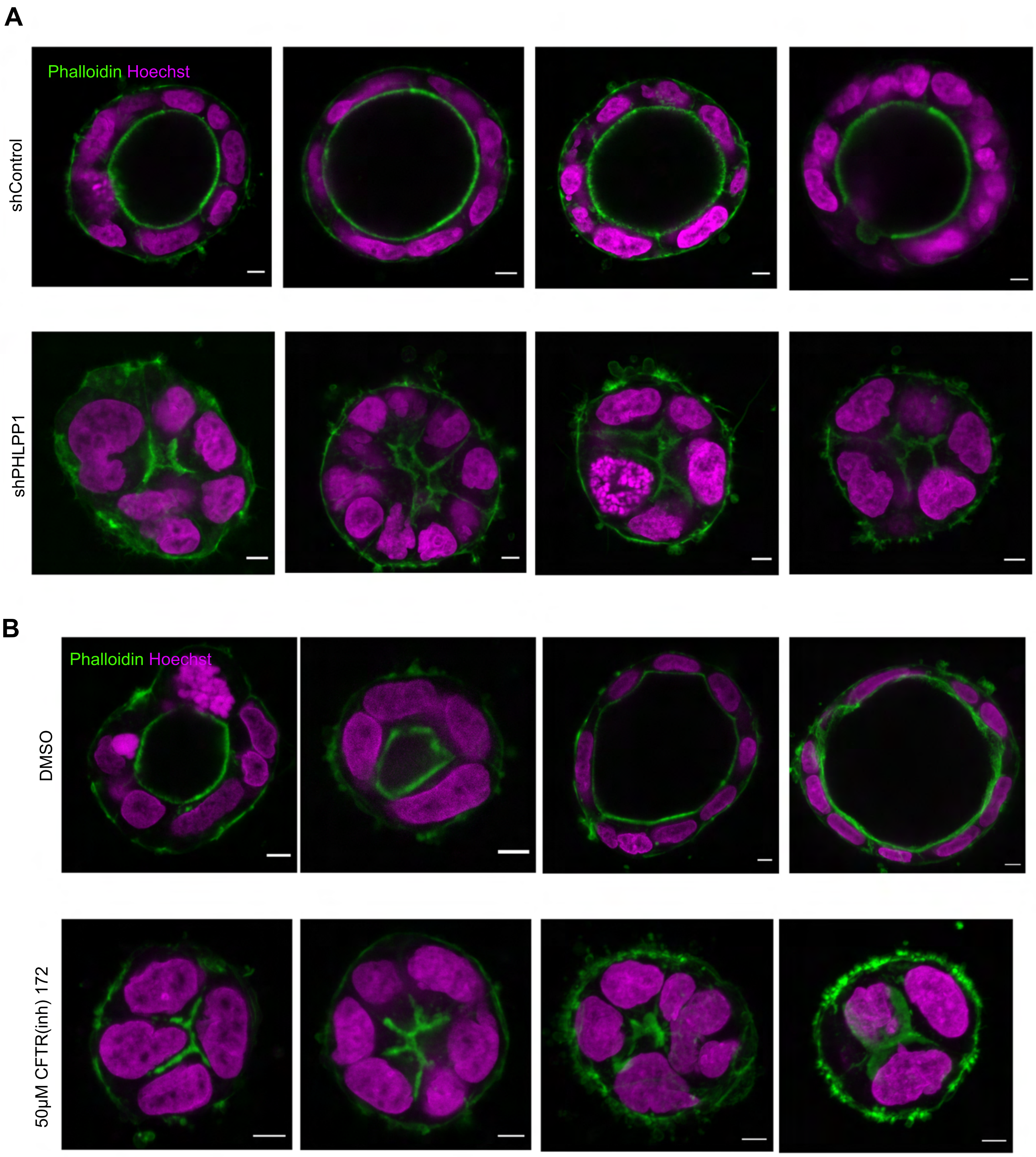
A
Phalloidin Hoechst
shControl
shPHLPP1
B
Phalloidin Hoechst
DMSO
50µM CFTR(inh) 172

Fig. S2. (A) Caco-2 cells expressing short hairpin RNA (shRNA) either non- targeting (shControl) or targeting PHLPP1 (shPHLPP1) were embedded in matrigel and collagen and labelled with phalloidin (green) and Hoechst dye 33342 (magenta). Scale bar, 5µm. (B) Caco-2 cells were embedded in matrigel and collagen and treated with DMSO or 50µM CFTR inhibitor-172 for a period of 72h. Cysts were labelled with phalloidin (green) and Hoechst dye 33342 (magenta). Scale bar, 5µm.

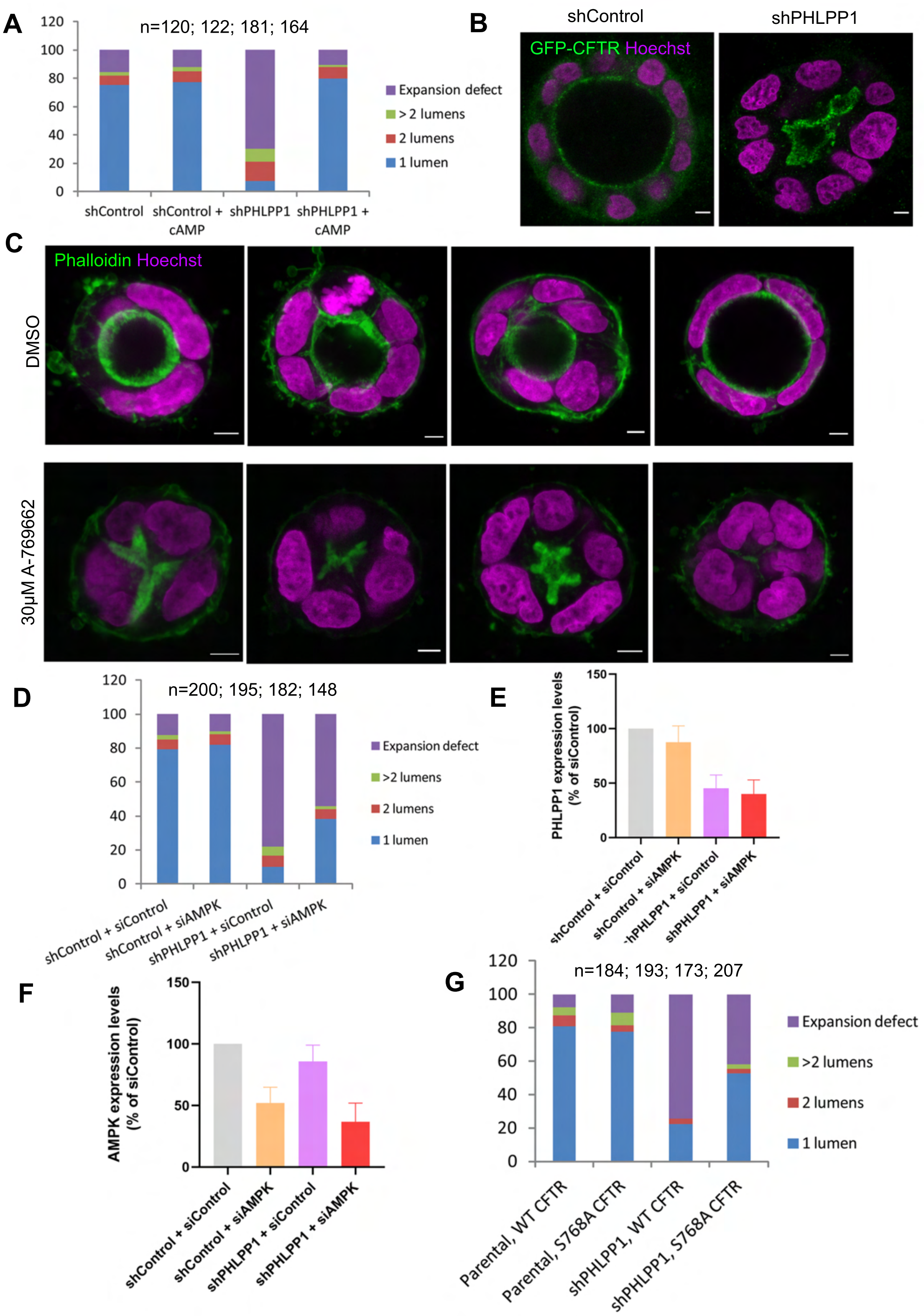
A
n=120; 122; 181; 164
120
100
80
60
40
20
0
shControl
shControl + cAMP
shPHLPP1
shPHLPP1 + cAMP
Expansion defect
> 2 lumens
2 lumens
1 lumen
B
shControl
shPHLPP1
GFP-CFTR Hoechst
C
Phalloidin Hoechst
DMSO
30µM A-769662
D
n=200; 195; 182; 148
shControl + siControl
shControl + siAMPK
shPHLPP1 + siControl
shPHLPP1 + siAMPK
Expansion defect
>2 lumens
2 lumens
1 lumen
E
PHLPP1 expression levels (% of siControl)
150
100
50
0
F
AMPK expression levels (% of siControl)
G
n=184; 193; 173; 207
Parental, WT CFTR
Parental, S768A CFTR
shPHLPP1, WT CFTR
shPHLPP1, S768A CFTR

Fig. S3. (A) Caco-2 expressing shRNA non-targeting or targeting PHLPP1 were embedded in matrigel and incubated with 100µM 6-Bnz-cAMP. These were scored as in Figure S1A. *n* represents the total number of Caco-2 cysts analysed in three independent experiments and is indicated in the graphs. (B) Caco-2 cells expressing GFP-CFTR were embedded in extracellular matrix and labelled with anti-GFP (green) and Hoechst 33342 dye (magenta). Scale bar, 5µm. (C) Caco-2 cells were embedded in matrigel and collagen and treated with DMSO or 30µM A-769662 for a period of 72h. Cysts were labelled with phalloidin (green) and Hoechst dye 33342 (magenta). Scale bar, 5µm. (D) Caco-2 cells expressing shRNA non- targeting or shPHLPP1 were transfected with siRNA targeting Control or AMPK, embedded in matrigel and collagen, and scored as in Figure S1A. *n* represents the total number of Caco-2 cysts analysed in three independent experiments and is indicated in the graphs. (E,F) Mean of PHLPP1 and AMPK expression levels was determined from relative signal intensities of PHLPP1 and AMPK bands following expression of shRNA targeting PHLPP1 or siRNA targeting AMPK. Control values were normalised to 1. Error bars represent s.e.m. (G) Control or shPHLPP1 Caco-2 cells stably expressing CFTR WT or CFTR S768A were embedded in matrigel and scored as per Fig. S1A. *n* represents the total number of Caco-2 cysts analysed in three independent experiments and is indicated in the graphs.
