# Supplementary material for: PHLPP1 regulates CFTR activity and lumen expansion through AMPK
Source: Development. 2022 Aug 23;149(20):dev200955. doi: 10.1242/dev.200955 (PMC9534488; doi:10.1242/dev.200955)
Supplement: Reviewer comments [file develop-149-200955_review_history.pdf]

PHLPP1 regulates CFTR activity and lumen expansion through AMPK

Viola H. Lobert, Maren L. Skardal, Lene Malerød, Julia E. Simensen, Hermine A. Algra, Aram N. Andersen, Thomas Fleischer, Hilde A. Enserink, Knut Liestøl, Joan K. Heath, Tor Erik Rusten and Harald A. Stenmark
DOI: 10.1242/dev.200955

Editor: Thomas Lecuit

Review timeline

Original submission:	23 August 2019
Editorial decision:	30 October 2019
Resubmission:	16 May 2022
Editorial decision:	21 June 2022
First revision received:	1 July 2022
Accepted:	12 July 2022

Original submission

First decision letter

MS ID#: DEVELOP/2019/184176

MS TITLE: PHLPP1 regulates lumen expansion through regulation of CFTR activity

AUTHORS: Viola H Lobert, Hilde A Enserink, Knut Liestøl, Tor Erik Rusten, Joan Heath, and Harald Stenmark

Dear Dr. Lobert,

I have now received all the referees' reports on the above manuscript, and have reached a decision. I am sorry to say that the outcome is not a positive one. The referees' comments are appended below, or you can access them online: please go to Development's submission site and click on the 'Manuscripts with Decisions' queue in the Author Area.

As you will see, the referees raise some significant concerns about your paper, and are not strongly in favour of publication. Having looked at the manuscript myself, I agree with their views, and I must therefore, reject your paper.

I do realise this is disappointing news, but Development receives many more papers than we can publish, and we can only accept manuscripts that receive strong support from referees.

I do hope you find the comments of the referees helpful, and that this decision will not dissuade you from considering Development for publication of your future work. Many thanks for sending your manuscript to Development.

Yours sincerely

Thomas Lecuit
Handling Editor
Development

© 2022. Published by The Company of Biologists under the terms of the Creative Commons Attribution License (https://creativecommons.org/licenses/by/4.0/).

Reviewer 1 Advance Summary and Potential Significance to Field:
In the manuscript by Lobert et al. the authors investigate the role of phosphatase PHLPP1 in regulating CFTR activity in Caco-2 3D cultures via AMPK. They find that depletion of PHLPP1 blocks lumen expansion in Caco-2 cysts due to activation of AMPK, a known negative effector of the CFTR channel. The data suggest PHLPP1 might control via AMPK regulation CFTR activity.

Reviewer 1 Comments for the Author:
This is a compact and straight forward study that identifies an interesting CFTR effector that might be of in vivo relevance and therefore could prompt further investigation. The manuscript is well written and is easy to follow. Overall, the experiments presented are logically designed and address immediate issues well.
However, the experimental design is a bit simplistic and could use more in-depth analyses. This may be achieved for example in one of two following ways: 1-For a cell culture study related to CFTR electrophysiological (e.g. patch or Ussing chamber data) characterization of channel properties is customary.
However, this may be outside the area of expertise of the authors. 2-Some sort of in vivo data implicating PHLPP1 or its downstream targets (i.e. AMPK or the phosphorylation site on CFTR), e.g. lumen expansion or fluid secretion in a biological tube. For Development one would expect to find in vivo or ex vivo data.

Specific points

1-In all cases the authors always show only one cyst. Including a lower mag image in the figures or as supplemental data would allow readers to better discern the consistency of the experiments.

2-Fig. 1C, can this be quantified?

3-The result of PHLPP2 depletion, seemingly distinct from that of PHLPP1 depletion is potentially interesting.
Is there any data suggesting different in vivo roles?

4-The authors examined CFTR localization and concluded it is not affected by PHLPP1 depletion or PKA-dependent activation. However, it is known that in cell lines and in the intestine PKA activation promotes CFTR surface localization (e.g. PMID:23868408, PMID:28592587). 4A-Is the GFP construct (not clear how it was generated) functional? 4B-Co-lableing of the apical membrane would help exclude that the CFTR signal corresponds to subapical vesicles.

5-Fig.3B, which CFTR bands (i.e. A, B, C) are present? Please indicate.

Minor points

6-Calling Caco-2 cysts “colon cysts” seems a bit of a stretch as this is a cell line and not a primary culture.

Reviewer 2 Advance Summary and Potential Significance to Field:
In this article, Lobert and co-workers have characterized a role for the tumor-suppressive phosphatase PHLPP1 in inactivating AMPK during lumen expansion. The authors observed lumen expansion defects upon PHLPP1 depletion, which were rescued by pharmacological activation of CFTR, and by reducing AMPK levels, or by expressing CFTR that cannot be phosphorylated by AMPK (S768A), suggesting that PHLPP1 regulates CFTR through AMPK.
They concluded that PHLPP1 modulates CFTR activity and lumen expansion.
Even though the message is compelling, the quality of the data and figures is rather poor, and it would need some modifications before being accepted for publication in Development.

Reviewer 2 Comments for the Author:
In this article, Lobert and co-workers have characterized a role for the tumor-suppressive phosphatase PHLPP1 in inactivating AMPK during lumen expansion. The authors observed lumen expansion defects upon PHLPP1 depletion, which were rescued by pharmacological activation of CFTR, and by reducing AMPK levels, or by expressing CFTR that cannot be phosphorylated by AMPK (S768A), suggesting that PHLPP1 regulates CFTR through AMPK.

© 2022. Published by The Company of Biologists under the terms of the Creative Commons Attribution License (https://creativecommons.org/licenses/by/4.0/).

They concluded that PHLPP1 modulates CFTR activity and lumen expansion.
Even though the message is compelling, the quality of the data and figures is rather poor, and it would need some modifications before being accepted for publication in Development.

1-The authors claim there is an effect with the CFTR inhibitor in the expansion of the lumen, but the procedure to quantify this effect is not clear to me. What are they measuring? The scale that goes from 0 to 4, and they state that control is adjusted to 1. There is no quantification on the effect of the AMPK activator on P-AMPK (Figure 1C). The staining of the spheroids with polarity markers is minimal, and thus gives little information on the polarization state of the cells. I would recommend using additional polarity markers to show how these treatments affect the polarization and differentiation state of the cells (i.e., junctions, apical markers, etc.)

2-Again in Figure 2, the silencing of PHLPP1 and two should be appropriately quantified with the statistical analysis in fugues 2b and 2f. The statistical analysis is also missing, or not represented in the figure, in the quantification of the effect of silencing these proteins in lumen expansion. There is an apparent minor effect of silencing PHLPP2 in Caco-2 cells morphogenesis, what is the effect on lumen expansion/formation of silencing PHLPP1 and PHLPP2 simultaneously? Could PHLPP1 be rescued by PHLPP2?

3-The effect of PHLPP1 on CFTR endogenous expression should be properly quantified with the statistical analysis in fugues 3b. Normally treatment with cAMP activators usually produces a very important effect on the expansion of the lumen in spheroids of Caco2 cells. However, no effect is seen in the experiment described in Figure 3d. Indeed, the lumens of spheroids treated with this drug look quite similar to controls (figure 3)

4-P-AMPK should also be quantified in the silencing of PHLPP2 (figures 4a and b). IP analysis of PHLPP1 and CFTR is inconclusive, and it requires more controls and quantifications. The silencing of AMPK in figure 4 e-f is not adequately quantified, but it seems to be very low. The authors should use different strategies to silence AMPK properly and evaluated the effect of Caco2 spheroid morphogenesis.

5-It is not clear to this reviewer how many cells are expressing the constructs WT CFTR and CFTR S768A since there in quantification or images showing the expression of these proteins. Indeed, this is also the case for many experiments along with the manuscript, and it is difficult to establish the number of cells that receive the siRNA/shRNA for silencing, etc.

Minor point
In line 120, the authors should include that “expression of CFTR S768A in PHLPP1-depleted cells PARTIALLY rescued the lumen expansion defects” since there is no complete recovery of the normal phenotype

Reviewer 3 Advance Summary and Potential Significance to Field:
This is a short and straight forward report describing the regulation of CFTR, a chroride channel involved in cystic fibrosis progression, in lumen formation. The authors use colorectal adenocarcinoma cells in matrigel cultures as their model for cyst formation. Previous published work had shown that PKA phosphorylates and activates CFTR whereas AMPK phosphorylates CFTR on a different Serine to inactivate it. This CFTR activity balance determines chlorine flux into the cells and influences cystic lumen expansion, presumably by modulating cellular osmosis and cell stretching.

The authors focus on how dephosphorylation of AMPK by PHLPP1/2 may modulate AMPK a and CFTR activity and first show that chemical inhibition of CFTR or activation of AMPK cause similar defects in cyst formation. Similarly, shRNA knockdown of PHLPP1, but not PHLPP2 disturb cyst expansion. This defect can be ameliorated by stimulation of AMPK by a cAMP analog suggesting that PHLPP1 and AMPK have antagonistic functions in cyst lumen growth. This is supported by the phenotypes of double knockdown of AMPK and PHLPP1 and by Western blots showing increased phosphorylation of MAPK upon PHLPP1 inhibition. Finally, a CFTR construct lacking the AMPK phosphorylation site rescues the shPHLPP1 defects in cystic lumen expansion suggesting that PHLPP1 controls CFTR activity.

© 2022. Published by The Company of Biologists under the terms of the Creative Commons Attribution License (https://creativecommons.org/licenses/by/4.0/).

Reviewer 3 Comments for the Author:

Overall the experiments are well controlled and presented. The paper is focusing on the regulatory circuit of CFTR activity but I believe that the readership of Development would appreciate the addition of a more detailed final drawing and discussion on how the activity of CFTR may influence cystic lumen formation.

Author response to reviewers' comments

Reviewer 1 suggests improving the manuscript in one of two following ways:
- Using an Ussing chamber
- Including in vivo data

I spent two weeks in the laboratory of Professor Jeffrey Beekman setting up Ussing chamber experiments using shControl Caco-2, shPHLPP1 Caco-2, GFP-CFTR shControl Caco-2 and GFP-CFTR shPHLPP1 Caco-2 cells. Unfortunately, the institute closed due to Covid-19 before I was able to make Ussing chamber measurements. We therefore performed some in vivo experiments in Drosophila instead.

There is only one Phlpp in Drosophila melanogaster. Importantly, a recent paper identified a Drosophila isoform for Cftr (PMID: 32345720), which plays a role in chloride transport in the gut. We therefore depleted Drosophila Phlpp in gut enterocytes and measured chloride using MQAE, a fluorescent indicator for intracellular chloride (see below, Fig. 1A-C). We did not observe a significant difference in chloride in guts where Phlpp was depleted (see below, Fig. 1A,B), while Cftr depletion resulted, as expected, in reduced chloride levels (see below, Fig. 1A,B). This data is currently not included in the manuscript. Additional in vivo data includes the analysis of mitotic spindle orientation in Drosophila neuroblasts (Fig. 2E-G), where we found that Drosophila Phlpp recapitulates human PHLPP2 function and plays a role in correct spindle orientation in metaphase cells.

Figure 1 will be visible in Supplementary Information.
Figure 1. (A) Fluorescent monitoring of Cl- using MQAE in Control, cftr RNAi or phlpp RNAi enterocytes of the midgut epithelium. Scale bar, 10µm. (B) Violin plot representing MQAE intensity measurements in Cftr RNAi midgut. Two-way ANOVA p-value comparing cftr RNAi to Control RNAi was 0.026. 10 intestines per cross were dissected, and 3 independent crosses were performed. (C) Violin plot representing MQAE intensity measurements in phlpp RNAi #1 midgut. Two-way ANOVA p-value comparing phlpp RNAi #1 to Control RNAi was 0.22. n is the number of midguts dissected, which was the result of three independent crosses.
NP-1-Gal4 flies (w; NP-1-Gal4/CyO) were kindly provided by Pankaj Kapahi, Buck Institute, USA. Cftr RNAi (w118P{GD238}v1204, 1204) was obtained from Vienna Drosophila Resource Centre (VDRC, Vienna Biocenter Core Facilities GmbH (VBCF), Austria). Knockdown of Phlpp in intestinal enterocytes was performed by crossing virgins of the enterocyte driver NP-1 with Phlpp RNAi males. Knockdown or Cftr by RNAi in intestinal enterocytes was performed by crossing Cftr RNAi virgins with NP-1 males.

Additionally, reviewer 1 has the following specific points.

1. “In all cases the authors always show only one cyst. Including a lower mag image in the figures or as supplemental data would allow readers to better discern the consistency of the experiments.”
We have now made Supplemental figure 2 and 3 with 4 additional pictures of shPHLPP1 cysts (Fig. S1A), CFTR inh-treated cysts (Fig. S2B), AMPK activator-treated cysts (Fig. S3C), which exemplify a consistent lack of lumen expansion in the different conditions compared to their respective controls (shControl and DMSO respectively). We have also indicated the number of cysts analysed on all the graphs.

2. “Fig. 1C, can this be quantified?”
We have now included a quantification of the increase of pAMPK/tAMPK upon treatment with the AMPK activator A-769662 in the new Figure 3F.

© 2022. Published by The Company of Biologists under the terms of the Creative Commons Attribution License (https://creativecommons.org/licenses/by/4.0/).

3. “The result of PHLPP2 depletion, seemingly distinct from that of PHLPP1 depletion is potentially interesting. Is there any data suggesting different in vivo roles?”
Recent evidence from the Trotman lab (PMID: 31092557) has found that PHLPP2 promotes prostate cancer progression, where it is upregulated in tumours lacking p53 and PTEN, and dephosphorylates Myc. In contrast, PHLPP1 deletion showed neoplastic lesions in prostate and colorectal tissue in mice (PMID: 21840483, PMID: 24530606). We do not currently have access to mouse models and only Drosophila melanogaster models. As indicated earlier, there is only one Phlpp in Drosophila, and we were able to observe that Drosophila Phlpp recapitulates human PHLPP2 function by regulating mitotic spindle orientation (Fig. 2F,G). In Caco-2 cysts, this role is important for the development of apical midbodies and a single lumen, which are disrupted in the absence of PHLPP2 (Fig. 2B,C,H,I).

4. “The authors examined CFTR localization and concluded it is not affected by PHLPP1 depletion or PKA- dependent activation. However, it is known that in cell lines and in the intestine PKA activation promotes CFTR surface localization (e.g. PMID:23868408, PMID:28592587). 4A-Is the GFP construct (not clear how it was generated) functional? 4B-Co-lableing of the apical membrane would help exclude that the CFTR signal corresponds to subapical vesicles.”

4A- The ENTR plasmids for eGFP-CFTR-WT and -S768A cDNAs were generated using the primers (mentioned in revised Materials and Methods) with traditional restriction enzyme-based cloning. Briefly, pcDNA3.1 CFTR-WT and -S768A were amplified using PCR from pcDNA3.1-CFTR WT and S768A plasmids (kindly gifted by Professor Kenneth Hallows) and were then cloned with NotI & XhoI sites in pENTR vector containing N-terminus HA-eGFP tags, which were verified with restriction enzymes cutting and sequencing. Next, expression vectors for stable cells were generated in pCDH-PGK-IRES vector using gateway LRII reaction mix, and these final expression vectors were again verified with restriction enzymes cutting and sequencing.
We placed GFP at the N-terminal of CFTR since this is reported to results in a functional CFTR. Addition of GFP to the N-terminus had no effect on localization, trafficking, and biophysical properties (PMID: 9705313).

5. “Fig.3B, which CFTR bands (i.e. A, B, C) are present? Please indicate”
We removed western blot experiments of CFTR from this manuscript as the antibody stopped working, and we were no longer able to detect CFTR protein levels. There is no evidence from imaging experiments that there is a difference in total protein levels, so this should not affect the conclusions of the manuscript.

6. “Calling Caco-2 cysts “colon cysts” seems a bit of a stretch as this is a cell line and not a primary culture. ”
The use of “colon cysts” on line 74 and in the figure legend for Figure 1 has been corrected to “Caco-2 cysts”.

Reviewer 2
1. “The authors claim there is an effect with the CFTR inhibitor in the expansion of the lumen, but the procedure to quantify this effect is not clear to me. What are they measuring? The scale that goes from 0 to 4, and they state that control is adjusted to 1. There is no quantification on the effect of the AMPK activator on P-AMPK (Figure 1C). The staining of the spheroids with polarity markers is minimal, and thus gives little information on the polarization state of the cells. I would recommend using additional polarity markers to show how these treatments affect the polarization and differentiation state of the cells (i.e., junctions, apical markers, etc.) ”
For Figure 1B, we have analysed Caco-2 cysts that have been incubated with 50μM CFTR inhibitor for a period of 72 hours. These were scored according to the categories 1 lumen, ≥2 lumens, expansion defects, single cells, 2 cell stage, blebbing cells, intracellular lumens. An average of 16.4% cysts in the DMSO control showed lumen expansion defects, while 42.9% of cysts treated with CFTR inhibitor showed expansion defects. We chose to normalise each control value to 1 in the initial manuscript, but have now replaced this with percentage (%) of cysts instead, and present the results in a violin plot (new Figure 3B).
For Figure 1C, we have now included a quantification of the effect of the AMPK inhibitor on pAMPK/tAMPK levels (new Figure 3F).

© 2022. Published by The Company of Biologists under the terms of the Creative Commons Attribution License (https://creativecommons.org/licenses/by/4.0/).

New immunofluorescence images have been added where markers such as E-cadherin and aPKC have been included (new Supplementary Figure 1C).

2. “Again in Figure 2, the silencing of PHLPP1 and two should be appropriately quantified with the statistical analysis in fugues 2b and 2f. The statistical analysis is also missing, or not represented in the figure, in the quantification of the effect of silencing these proteins in lumen expansion. There is an apparent minor effect of silencing PHLPP2 in Caco-2 cells morphogenesis, what is the effect on lumen expansion/formation of silencing PHLPP1 and PHLPP2 simultaneously? Could PHLPP1 be rescued by PHLPP2?”
The efficiency of silencing the expression of PHLPP1 by siRNA and shRNA has now been quantified and added to the figure (new Supplemental Figure 1D,E,F).
The p-values for the effects of the silencing of PHLPP1 on lumen expansion were indicated in the Figure legend 2, and asterisks indicating significance have also now been added to the following figures for clarity (new Figure 1C,D).
Silencing of PHLPP2 results in a significant increase in cysts containing multiple lumens (Fig. 1B,D). Silencing PHLPP1 and PHLPP2 simultaneously leads to lumen expansion defects and multiple lumens (new Fig. E,F). The phenotypes resulting from depletion from PHLPP1 or PHLPP2 are therefore distinct.

3. “The effect of PHLPP1 on CFTR endogenous expression should be properly quantified with the statistical analysis in fugues 3b. Normally treatment with cAMP activators usually produces a very important effect on the expansion of the lumen in spheroids of Caco2 cells. However, no effect is seen in the experiment described in Figure 3d. Indeed, the lumens of spheroids treated with this drug look quite similar to controls (figure 3) 4-P-AMPK should also be quantified in the silencing of PHLPP2 (figures 4a and b). IP analysis of PHLPP1 and CFTR is inconclusive, and it requires more controls and quantifications. The silencing of AMPK in figure 4 e-f is not adequately quantified, but it seems to be very low. The authors should use different strategies to silence AMPK properly and evaluated the effect of Caco2 spheroid morphogenesis.”

As mentioned, the CFTR antibody stopped working on Western blot and we therefore had to remove this data from the manuscript.
cAMP activators absolutely accelerate lumen expansion. However, we analysed our cysts after 5 days in matrigel, once lumen expansion is complete. We therefore do not expect differences at this time-point. There are surely differences in lumen expansion rates earlier on.
Quantification of pAMPK in siPHLPP1 lysates is now indicated in new Fig. 4B.
Silencing of AMPK is now quantified in a new Supplemental Figure 3F. The reason for depleting AMPK partially is so that its depletion does not result in phenotypes of its own. Indeed, depletion of AMPK would result in mitotic spindle orientation defects and multiple lumens as well as other phenotypes if completely silenced. The aim of this experiment is to reduce the amount of AMPK that is hyperactivated when PHLPP1 is absent.

4. “It is not clear to this reviewer how many cells are expressing the constructs WT CFTR and CFTR S768A since there in quantification or images showing the expression of these proteins. Indeed, this is also the case for many experiments along with the manuscript, and it is difficult to establish the number of cells that receive the siRNA/shRNA for silencing, etc.”
This was an omission in the submitted manuscript and we apologise. The numbers of cysts expressing WT CFTR or CFTR S768A that were analysed for lumen formation are now indicated in the graph (new Figure 4G). Images of GFP-CFTR and CFTR S768A are now included in new Figure 4F. The penetrance of the lumen expansion phenotype is high upon expression of shRNA targeting PHLPP1 (new Fig. 1F). Efficiency of PHLPP1 knock-down upon expression of shRNA is now included in new Supplemental Figure 1F,G. This suggests most cells have depleted PHLPP1. With the case of GFP-expressing cells, we used adenovirus to make stable lines expressing GFP-CFTR WT and S768A. GFP-positive cells were selected by flow cytometry, so only GFP-expressing cells were analysed.

“Minor point
In line 120, the authors should include that “expression of CFTR S768A in PHLPP1-depleted cells PARTIALLY rescued the lumen expansion defects” since there is no complete recovery of the normal phenotype”

This has been corrected in the new manuscript file.

© 2022. Published by The Company of Biologists under the terms of the Creative Commons Attribution License (https://creativecommons.org/licenses/by/4.0/).

Reviewer 3

“Overall the experiments are well controlled and presented. The paper is focusing on the regulatory circuit of CFTR activity but I believe that the readership of Development would appreciate the addition of a more detailed final drawing and discussion on how the activity of CFTR may influence cystic lumen formation.”
The graphical abstract that had been included in the cover letter has now been placed in new Figure 5D.

Resubmission

First decision letter

MS ID#: DEVELOP/2022/200955

MS TITLE: PHLPP1 regulates CFTR activity and lumen expansion through AMPK

AUTHORS: Viola H Lobert, Maren L Skardal, Lene Malerød, Julia E Simensen, Hermine A Algra, Aram N Andersen, Thomas Fleischer, Hilde A Enserink, Knut Liestøl, Joan K Heath, Tor Erik Rusten, and Harald A Stenmark

I have now received all the referees reports on the above manuscript, and have reached a decision. The referees' comments are appended below, or you can access them online: please go to BenchPress and click on the 'Manuscripts with Decisions' queue in the Author Area.

The overall evaluation is very positive and we would like to publish a revised manuscript in Development, provided that the referees' final minor comments are satisfactorily addressed.

Reviewer 1

Advance summary and potential significance to field

The authors have revised their original manuscript to address concerns raised by the reviewers. With the inclusion of new data and various revisions of previously existing figures the authors have improved quantitations and expanded the in vivo relevance of their findings. Overall, the manuscript seems significantly improved and provides useful insights into CFTR regulation that should be interesting for a broad field.

Comments for the author

If further text revisions are made, the authors should consider referencing the fact that CFTR also transport bicarbonate as this is likely the main factor affecting mucin secretions.

Reviewer 2

Advance summary and potential significance to field

In the manuscript entitled "PHLPP1 regulates CFTR activity and lumen 1 expansion through AMPK" the authors talk about the importance of PHLPP1 in inhibiting AMPK which helps to keep CFTR open and functioning at the apical membrane aiding in lumenogenesis. This paper is a revision to a previous submission and the authors address the previous concerns thoroughly. The paper was a pleasure to read and this new reviewer enjoyed the comparison with spindle orientation and CFTR function as two separate mechanisms PHLPP1 can be involved in during lumenogenesis and provides significant advancement to the field. They not only worked with CACO-2 cells with all relevant

© 2022. Published by The Company of Biologists under the terms of the Creative Commons Attribution License (https://creativecommons.org/licenses/by/4.0/).

controls, but they also further showed that PHLPP is involved in neuroblast spindle positioning in drosophila demonstrating a breadth and depth to their studies.
Based on their thorough rebuttal letter to the previous reviews and my assessment of the current manuscript I suggest that this study should be accepted for publication at Development.

Comments for the author

I would encourage acceptance with minor text revision. Comments included below:
- Figure 5D in the figure legend is not the same as what figure 5D shows.
- In the main text, there is no description or mention of Figure 5D-G of the figures shown
- In figures S3B-C, the green channel is said to be GFP. The figure legend and the text say it is GFP-CFTR.
- Expand upon why CACO-2 was the chosen cell model for these studies over other models.

First revision

Author response to reviewers' comments

Response to reviewers' comments

Reviewer 1 Comments for the Author:
If further text revisions are made, the authors should consider referencing the fact that CFTR also transport bicarbonate as this is likely the main factor affecting mucin secretions.

We thank the reviewer for this valuable comment and have implemented the following changes:
Line 21: added bicarbonate
Line 203-4: added reference Chen, E. Y., et al. (2010). "A new role for bicarbonate in mucus formation." Am J Physiol Lung Cell Mol Physiol **299**(4): L542-549.
Line 527-30: added bicarbonate three times

Reviewer 2 Comments for the Author:
I would encourage acceptance with minor text revision. Comments included below:

•Figure 5D in the figure legend is not the same as what figure 5D shows.

The text describing the cartoon has now been modified to take into account this comment.

•In the main text, there is no description or mention of Figure 5D-G of the figures shown

I assume the reviewer means Figure 4, since there is no Figure 5E-G. On line 171, Fig. 4C-E is referred to, and on line 175 Fig. 4F,G is referred to.

•In figures S3B-C, the green channel is said to be GFP. The figure legend and the text say it is GFP- CFTR.

This has now been corrected to GFP-CFTR in Fig. S3B and to Phalloidin in Fig. S3C.

•Expand upon why CACO-2 was the chosen cell model for these studies over other models

We have inserted information on this point on lines 69-72.

© 2022. Published by The Company of Biologists under the terms of the Creative Commons Attribution License (https://creativecommons.org/licenses/by/4.0/).

Second decision letter

MS ID#: DEVELOP/2022/200955

MS TITLE: PHLPP1 regulates CFTR activity and lumen expansion through AMPK

AUTHORS: Viola H Lobert, Maren L Skardal, Lene Malerød, Julia E Simensen, Hermine A Algra, Aram N Andersen, Thomas Fleischer, Hilde A Enserink, Knut Liestøl, Joan K Heath, Tor Erik Rusten, and Harald A Stenmark
ARTICLE TYPE: Research Article

I am happy to tell you that your manuscript has been accepted for publication in Development, pending our standard ethics checks.

© 2022. Published by The Company of Biologists under the terms of the Creative Commons Attribution License (https://creativecommons.org/licenses/by/4.0/).
